# Association of serum vascular endothelial growth factor-C, vascular endothelial growth factor receptor-3, and insulin-like growth factor 1 levels with metastasis and prognosis in patients with nasopharyngeal carcinoma

**DOI:** 10.3389/fonc.2025.1655015

**Published:** 2025-11-17

**Authors:** Yongchun Li, Le Wang, Yutong Zhao, Jia Zhao, Wulin Wen

**Affiliations:** Department of Otolaryngology Head and Neck Surgery, Yinchuan First People’s Hospital, Otolaryngology Head and Neck Surgery Hospital, Yinchuan, Ningxia, China

**Keywords:** serum VEGFC, VEGFR-3, IGF1, nasopharyngeal carcinoma, metastasis, prognosis, EBV DNA, nomogram

## Abstract

**Objective:**

To investigate the association of serum levels of Vascular Endothelial Growth Factor-C (VEGFC), Vascular Endothelial Growth Factor Receptor-3 (VEGFR-3), and Insulin-like Growth Factor 1 (IGF1) with metastasis and prognosis in patients with nasopharyngeal carcinoma (NPC).

**Methods:**

This retrospective study included 298 patients diagnosed with NPC at our institution between January 2022 and December 2023. Patients were categorized based on the presence of metastasis at diagnosis or during follow-up into a metastatic group (n=78) and a non-metastatic group (n=220). Clinical data, including plasma Epstein-Barr virus (EBV) DNA load, were collected, and serum VEGFC, VEGFR-3, and IGF1 levels were measured. Patients were followed up for a mean of (12.02 ± 1.21) months (minimum 12 months). Univariate and multivariate logistic regression analyses were performed to identify factors influencing NPC metastasis. Receiver Operating Characteristic (ROC) curve analysis was used to evaluate the predictive value of these biomarkers for NPC metastasis. Kaplan-Meier survival analysis and log-rank tests were used to evaluate the prognostic value of the biomarkers for OS.

**Results:**

Serum levels of VEGFC, VEGFR-3, and IGF1 were significantly higher in the metastatic group compared to the non-metastatic group (P<0.05). Similarly, these markers were significantly elevated in patients with poor prognosis compared to those with good prognosis (P<0.05). Multivariate logistic regression analysis identified advanced T stage, N stage, high plasma EBV DNA load, and elevated serum VEGFC, VEGFR-3, and IGF1 levels as independent risk factors for NPC metastasis. Combined detection of serum VEGFC, VEGFR-3, and IGF1 yielded a significantly higher Area Under the Curve (AUC) for predicting NPC metastasis than individual markers, and a nomogram incorporating all independent risk factors showed excellent predictive performance (C-index: 0.941). Kaplan-Meier analysis revealed that patients with high levels of VEGFC, VEGFR-3, IGF1, or a high-risk score from the combined biomarker model had significantly poorer OS (all P<0.001).

**Conclusion:**

Serum levels of VEGFC, VEGFR-3, and IGF1 are significantly correlated with metastasis and poor prognosis in NPC patients. These biomarkers, particularly when combined and integrated with EBV DNA load, serve as valuable indicators for predicting metastatic risk and assessing survival outcomes in NPC.

## 1 Introduction

Nasopharyngeal carcinoma (NPC) is a malignancy arising from the epithelial lining of the nasopharynx, most commonly in the fossa of Rosenmüller (1). While relatively rare in many parts of the world, NPC exhibits a striking geographical distribution, with high incidence rates in Southeast Asia, Southern China, North Africa, and the Arctic (2). The etiology of NPC is multifactorial, involving genetic predisposition, environmental factors, and a strong association with Epstein-Barr virus (EBV) infection, which is considered a primary causative agent (3, 4).

According to the 8th edition of the American Joint Committee on Cancer (AJCC) staging system, patients with stage III and IV disease are classified as having advanced NPC. Stage III and IVA represent locally advanced disease, while stage IVB indicates metastatic disease (5). Due to the insidious onset and non-specific early symptoms, such as nasal obstruction, epistaxis, or hearing loss, approximately 70% of NPC patients present with locally advanced or metastatic disease at the time of diagnosis (6). While early-stage NPC boasts a 5-year survival rate of up to 90%, the 5-year survival rate for locally advanced NPC drops to 60-80% (7). Distant metastasis remains the primary cause of treatment failure and mortality in NPC patients (8). Therefore, identifying reliable biomarkers for early detection of metastasis and accurate prognostic assessment is crucial for optimizing treatment strategies and improving patient outcomes.

Vascular endothelial growth factor-C (VEGFC) is a key regulator of lymphangiogenesis, the formation of new lymphatic vessels. VEGFC exerts its effects by binding to its cognate receptor, vascular endothelial growth factor receptor-3 (VEGFR-3), predominantly expressed on lymphatic endothelial cells (9). The VEGFC/VEGFR-3 signaling pathway plays a critical role in tumor lymphangiogenesis, facilitating the dissemination of tumor cells to regional lymph nodes and distant sites (10). For instance, recent studies have demonstrated that enhanced VEGFC secretion promotes lymphangiogenesis and lymph node metastasis in various cancers, such as bladder cancer (11). Lymphangiogenesis, orchestrated by the VEGFC-VEGFR-3 axis and often mediated by immune cells like macrophages in the tumor microenvironment, is a pivotal step in lymphatic metastasis (12).

Insulin-like growth factor 1 (IGF1), a polypeptide hormone, is involved in regulating cell growth, proliferation, differentiation, and apoptosis. Aberrant IGF1 signaling has been implicated in the development and progression of various cancers (13). IGF1 can promote tumor cell invasion and metastasis by modulating cell adhesion and angiogenesis (14). Previous work reported a positive correlation between IGF1 concentrations and tumor size in NPC patients, suggesting its potential as a diagnostic marker (15). However, research on the combined roles of serum VEGFC, VEGFR-3, and IGF1 in relation to metastasis and prognosis specifically in the context of NPC remains relatively limited.

The rationale for selecting these three specific biomarkers is based on their distinct yet complementary roles in critical pathways of NPC progression. The VEGFC/VEGFR-3 axis is a well-established driver of lymphangiogenesis, a primary route for NPC metastasis. Concurrently, the IGF1 signaling pathway is integral to tumor cell proliferation, survival, and invasion. By investigating these markers in combination, we hypothesized that a multi-faceted biomarker panel targeting both lymphatic spread and tumor growth dynamics would provide a more comprehensive and powerful prognostic tool than markers from a single biological pathway. Given the critical roles of these molecules in tumor biology, this study aimed to retrospectively investigate the association of serum VEGFC, VEGFR-3, and IGF1 levels with metastasis and prognosis in a cohort of NPC patients. The findings could provide new insights for the prevention of NPC metastasis and offer hope for prolonging patient survival by identifying potential therapeutic targets and prognostic biomarkers.

## 2 Materials and methods

### 2.1 Study design and ethical approval

This retrospective cohort study was conducted in accordance with the ethical principles outlined in the Declaration of Helsinki. Approval for the study protocol was obtained from the Institutional Review Board and Ethics Committee of Yinchuan First People’s Hospital (Approval No. YFPH-2024045) on June 9, 2024, prior to the collection and analysis of retrospective data. Due to the retrospective nature of the study, the requirement for individual informed consent was waived by the ethics committee, provided that patient anonymity was maintained.

### 2.2 Patient enrollment and selection

**Figure 1:**
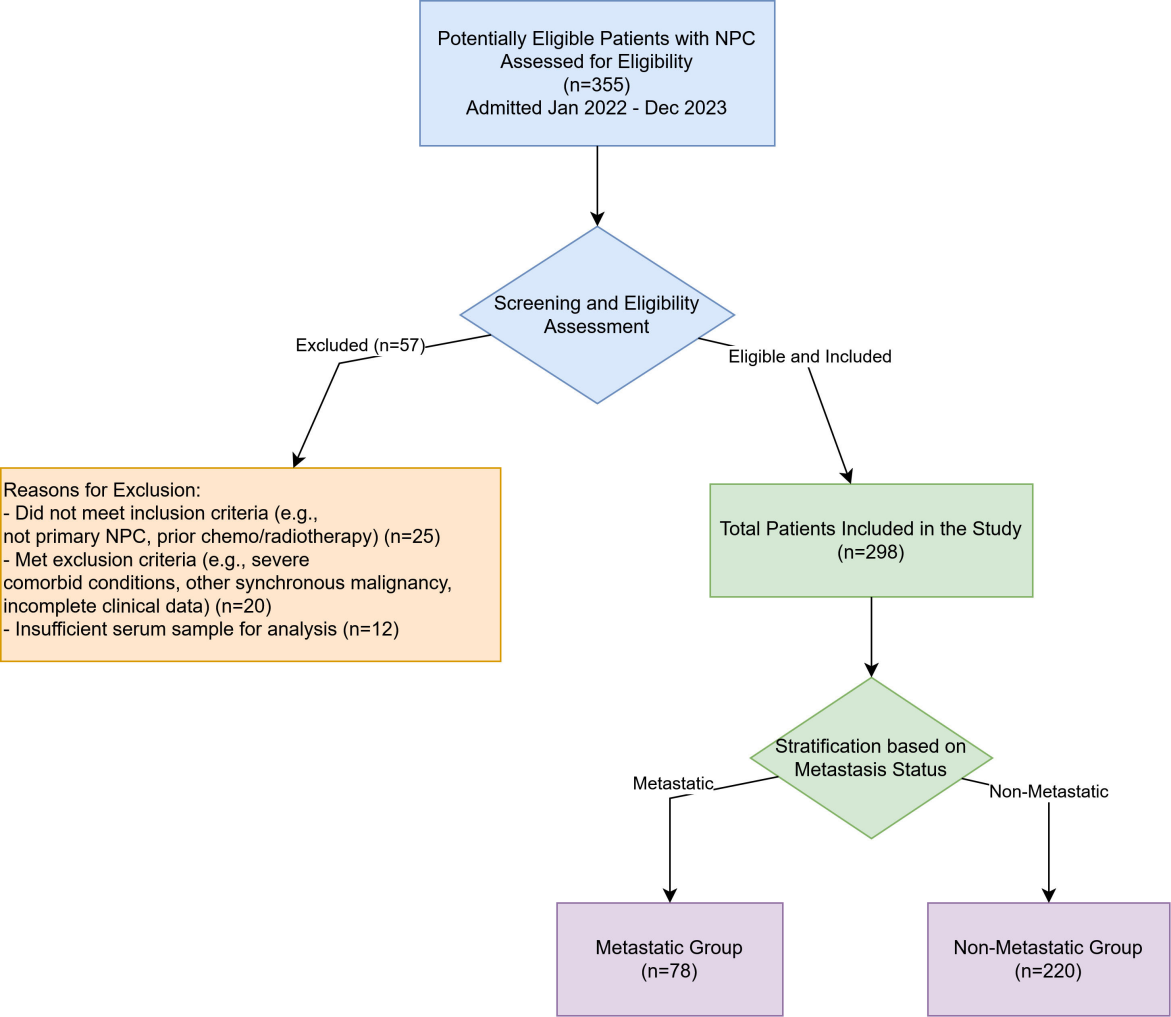
Flowchart of patient enrollment and selection. A total of 355 patients with nasopharyngeal carcinoma admitted between January 2022 and December 2023 were initially assessed for eligibility. Fifty-seven patients were excluded, resulting in 298 eligible patients who were included in the final analysis and subsequently stratified into the Metastatic Group (n=78) and the Non-Metastatic Group (n=220).

Patients diagnosed with NPC and admitted to our hospital between January 2022 and December 2023 were retrospectively identified from medical records. A systematic screening process was employed to determine eligibility for inclusion in the study. Initially, records of 355 patients with a diagnosis of NPC were assessed for eligibility. Of these, 57 patients were excluded: 25 did not meet specific inclusion criteria (e.g., diagnosis not pathologically confirmed as primary NPC, had received prior anti-cancer treatment such as chemotherapy or radiotherapy before baseline sample collection), 20 met exclusion criteria (e.g., presence of other synchronous malignancies, severe cardiac, hepatic, or renal dysfunction impacting prognosis or biomarker assessment, or had incomplete essential clinical data), and 12 had insufficient or improperly stored serum samples for reliable biomarker analysis. After this screening and eligibility assessment, a total of 298 patients were enrolled in the study and formed the basis for this analysis. The patient selection process is detailed in **Figure 1**.

### 2.3 Study population

A total of 298 NPC patients were included. Patients were divided into a metastatic group (n=78) and a non-metastatic group (n=220) based on the presence or absence of distant metastasis confirmed by imaging (e.g., CT, MRI, PET-CT) and/or pathological examination at the time of diagnosis or during follow-up.

#### 2.3.1 Inclusion criteria

(1) Pathologically confirmed diagnosis of NPC according to World Health Organization (WHO) criteria (16), verified by nasopharyngoscopy, magnetic resonance imaging (MRI), computed tomography (CT), and histopathology; (2) Newly diagnosed NPC patients without prior radiotherapy, chemotherapy, surgery, or biological therapy; (3) Estimated survival time >3 months; (4) Karnofsky Performance Status (KPS) score >60.

#### 2.3.2 Exclusion criteria

(1) Severe malnutrition or cachexia; (2) Concurrent malignant tumors other than NPC; (3) Severe cardiac, hepatic, or renal dysfunction; (4) History of previous anti-tumor therapy; (5) Presence of acute or chronic inflammation, acute infectious diseases, or hematological system disorders.

### 2.4 Data collection

Clinical data for all patients were collected from their medical records, including age, gender, disease duration, pathological type, clinical tumor stage (according to the 8th AJCC staging system), KPS score, smoking history, alcohol consumption, body mass index (BMI), and plasma Epstein-Barr virus (EBV) DNA load at diagnosis.

### 2.5 Serum biomarker and EBV DNA measurement

Fasting venous blood samples (5 mL) were collected from all study participants before any treatment. Samples were centrifuged at 3000 r/min for 10 minutes (centrifuge radius 10 cm) to separate the serum and plasma. The isolated serum and plasma were aliquoted and stored at -80°C until analysis to ensure sample stability. All samples were analyzed within 6 months of collection. Serum levels of VEGFC (Cat. No. Lz-M2154), VEGFR-3 (Cat. No. Lz-M3088), and IGF1 (Cat. No. Lz-M1897) were determined using enzyme-linked immunosorbent assay (ELISA) kits (Zhuhai LIZHU Reagent Co., Ltd., Zhuhai, China), strictly following the manufacturer’s instructions. All measurements were performed in duplicate. Plasma EBV DNA was quantified using a real-time quantitative polymerase chain reaction (qPCR) assay targeting the BamHI-W region of the EBV genome, with results reported in copies/mL. A value of ≥2000 copies/mL was defined as high EBV DNA load.

### 2.6 Follow-up and prognostic assessment

All patients were followed up for at least 1 year. Follow-up data were collected until January 2025, ensuring that all patients in the cohort, including those diagnosed in December 2023, had a minimum follow-up of 12 months. Patients were invited for review every 3 months. The primary endpoint for this study was the presence of distant metastasis. The secondary endpoint was Overall Survival (OS), defined as the time from diagnosis to death from any cause. Patients alive at the last follow-up were censored. For the purpose of this study’s primary analysis, prognosis was directly linked to metastatic status. Patients in the metastatic group (n=78) were categorized into the “poor prognosis group,” while those in the non-metastatic group (n=220) were categorized into the “good prognosis group,” as distant metastasis is the principal determinant of poor outcomes in NPC.

### 2.7 Statistical analysis

Statistical analyses were performed using SPSS version 28.0 (IBM Corp., Armonk, NY, USA) and R software version 4.2.1 (R Foundation for Statistical Computing, Vienna, Austria). Continuous data are presented as mean ± standard deviation (mean ± SD) if normally distributed, and differences between two groups were analyzed using an independent samples t-test. Non-normally distributed continuous data are presented as median (interquartile range, IQR), and comparisons were made using the Mann-Whitney U test. Categorical data are presented as counts (percentages) [n (%)] and compared using the chi-square (χ²) test or Fisher’s exact test, as appropriate. Univariate and multivariate logistic regression analyses were performed to identify independent predictors of NPC metastasis. A predictive nomogram was constructed based on the results of the multivariate logistic regression analysis using the “rms” package in R. The performance of the nomogram was evaluated by the concordance index (C-index) and calibration curves. Receiver Operating Characteristic (ROC) curve analysis was conducted to evaluate the diagnostic performance of serum biomarkers in predicting NPC metastasis, and the area under the curve (AUC) was calculated. For survival analysis, patients were dichotomized into “low” and “high” groups based on the optimal cut-off values for VEGFC, VEGFR-3, and IGF1 derived from the ROC analysis. The combined biomarker model was used to stratify patients into low-risk and high-risk groups. OS curves were generated using the Kaplan-Meier method, and differences were compared using the log-rank test. A P-value <0.05 was considered statistically significant. All p-values were reported consistently (e.g., P<0.001 or P = 0.012).

## 3 Results

### 3.1 Comparison of baseline characteristics

All 298 patients were followed up for a minimum of 1 year, with a mean follow-up duration of (12.02 ± 1.21) months. Based on metastatic status, patients were divided into the poor prognosis group (n=78) and the good prognosis group (n=220). There were no significant differences between the groups in terms of gender, smoking habits, alcohol consumption, BMI, or disease duration (P>0.05). However, significant differences were observed in age, clinical stage, T stage, N stage, and plasma EBV DNA load (P<0.05), as shown in **Table 1**.

**Table 1:** Comparison of baseline characteristics between poor and good prognosis groups.

Characteristic	Poor Prognosis Group (n=78)	Good Prognosis Group (n=220)	χ² value	P-value
Gender (Male, n (%))	56 (71.8)	158 (71.8)	0.000	0.998
Age >60 years, n (%)	45 (57.7)	81 (36.8)	9.865	0.002
Smoking (Yes, n (%))	28 (35.9)	75 (34.1)	0.076	0.783
Alcohol Consumption (Yes, n (%))	25 (32.1)	61 (27.7)	0.618	0.432
Pathological Type, n (%)			0.315	0.854
Undifferentiated non-keratinizing	53 (67.9)	154 (70.0)		
Differentiated non-keratinizing	21 (26.9)	57 (25.9)		
emspKeratinizing	4 (5.1)	9 (4.1)		
Clinical Stage (AJCC 8th Ed.), n (%)			18.551	<0.001
Stage III	20 (25.6)	152 (69.1)		
Stage IVA	58 (74.4)	68 (30.9)		
T Stage, n (%)			14.772	<0.001
T1-T2	21 (26.9)	118 (53.6)		
T3-T4	57 (73.1)	102 (46.4)		
N Stage, n (%)			19.638	<0.001
N0-N2	18 (23.1)	135 (61.4)		
N3	60 (76.9)	85 (38.6)		
EBV DNA Load (copies/mL), n (%)			16.213	<0.001
High (≥2000)	62 (79.5)	95 (43.2)		
Low (<2000)	16 (20.5)	125 (56.8)		

### 3.2 Comparison of serum biomarker levels between groups

Serum levels of VEGFC, VEGFR-3, and IGF1 were significantly higher in the poor prognosis/metastatic group (n=78) compared to the good prognosis/non-metastatic group (n=220). All differences were statistically significant (P<0.001), as detailed in **Table 2**. Furthermore, a correlation analysis was performed between the biomarkers and EBV DNA load. As shown in **Supplementary Table S1**, VEGFC, VEGFR-3, and IGF1 levels all showed a significant positive correlation with plasma EBV DNA load (all p < 0.001).

**Table 2:** Comparison of serum biomarker levels between poor and good prognosis groups (mean ± SD).

Biomarker	Poor Prognosis Group (n=78)	Good Prognosis Group (n=220)	t-value	P-value
VEGFC (ng/L)	545.29±46.34	472.17±39.41	-11.168	<0.001
VEGFR-3 (ng/L)	1276.37±97.63	1138.51±96.56	-8.818	<0.001
IGF1 (ng/mL)	112.78±17.65	47.22±11.32	17.603	<0.001

### 3.3 Univariate and multivariate analyses of factors associated with NPC metastasis

Univariate logistic regression analysis identified advanced T stage, N stage, high EBV DNA load, and elevated serum levels of VEGFC, VEGFR-3, and IGF1 as significant risk factors for NPC metastasis (**Table 3**). To avoid multicollinearity, variables with significant correlations were assessed before inclusion in the multivariate model. All significant factors from the univariate analysis were then included in a multivariate logistic regression model. The analysis confirmed that advanced T stage (OR = 4.512, P<0.001), N stage (OR = 5.981, P<0.001), high EBV DNA load (OR = 3.551, P = 0.002), and elevated serum levels of VEGFC (OR = 3.917, P<0.001), VEGFR-3 (OR = 1.503, P = 0.003), and IGF1 (OR = 2.418, P<0.001) were independent risk factors for NPC metastasis (**Table 4**).

**Table 3:** Univariate logistic regression analysis of factors associated with NPC metastasis.

Factor	β	SE	Wald χ²	OR (95% CI)	P-value
Age (>60 vs. ≤60 years)	0.875	0.339	6.654	2.400 (1.235–4.661)	0.010
T Stage (T3-T4 vs. T1-T2)	1.258	0.345	13.310	3.518 (1.792–6.908)	<0.001
N Stage (N3 vs. N0-N2)	1.698	0.354	22.956	5.461 (2.731–10.922)	<0.001
EBV DNA (High vs. Low)	1.493	0.366	16.632	4.450 (2.175–9.106)	<0.001
VEGFC (High vs. Low)	2.333	0.395	34.793	10.306 (4.757–22.327)	<0.001
VEGFR-3 (High vs. Low)	2.103	0.362	33.725	8.190 (4.026–16.662)	<0.001
IGF1 (High vs. Low)	2.845	0.430	43.766	17.202 (7.404–39.965)	<0.001

**Table 4:** Multivariate logistic regression analysis of factors associated with NPC metastasis.

Factor	β	SE	Wald χ²	OR (95% CI)	P-value
T Stage (T3-T4 vs. T1-T2)	1.507	0.435	11.975	4.512 (1.918–10.612)	<0.001
N Stage (N3 vs. N0-N2)	1.789	0.451	15.721	5.981 (2.481–14.421)	<0.001
EBV DNA (High vs. Low)	1.267	0.418	9.183	3.551 (1.567–8.049)	0.002
VEGFC (High vs. Low)	1.365	0.431	10.024	3.917 (1.681–9.129)	<0.001
VEGFR-3 (High vs. Low)	0.407	0.399	2.996	1.503 (1.006–3.294)	0.003
IGF1 (High vs. Low)	0.883	0.463	12.783	2.418 (1.572–5.945)	<0.001

### 3.4 ROC curve analysis and nomogram for predicting NPC metastasis

**Figure 2:**
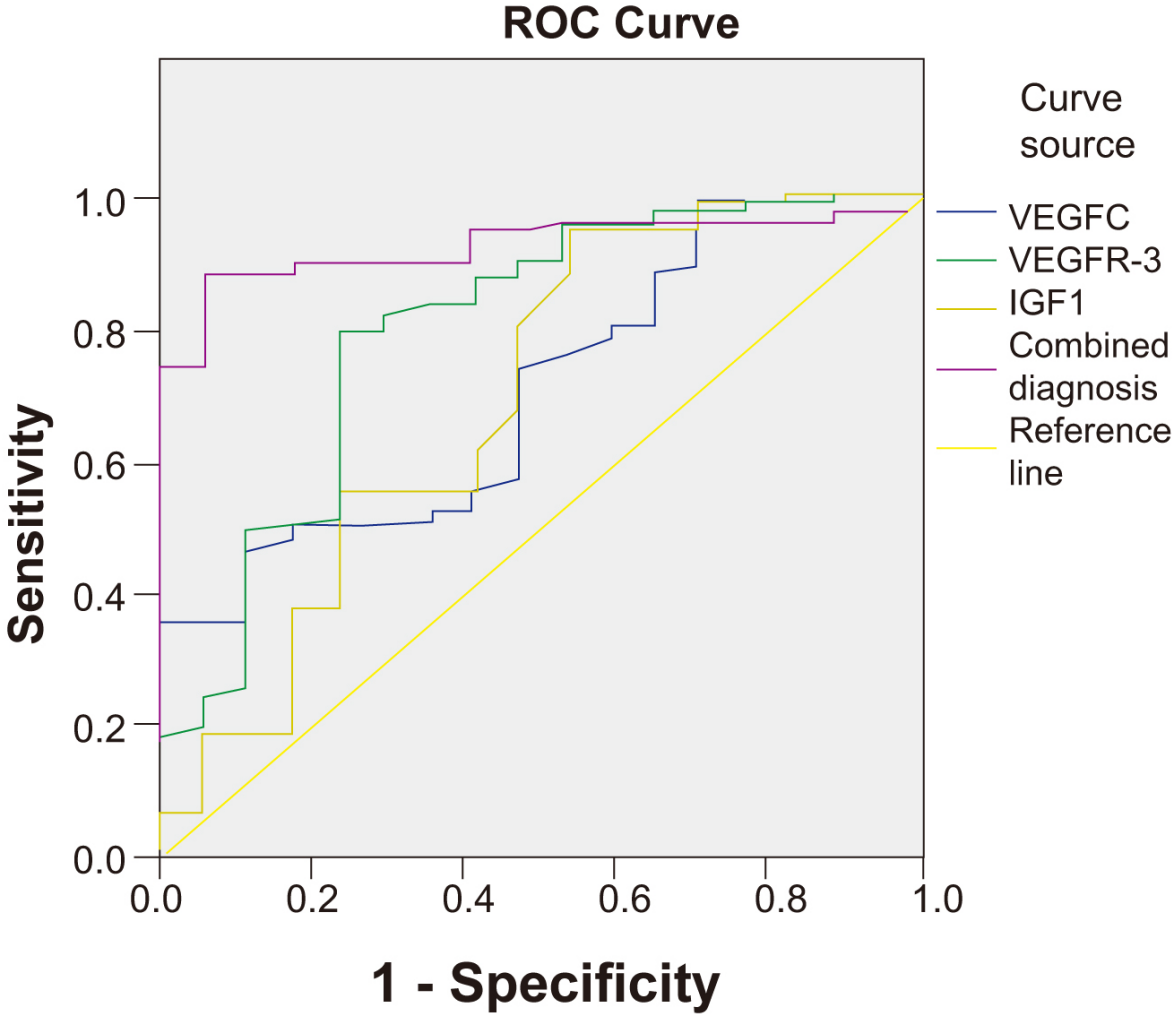
ROC curves for serum VEGFC, VEGFR-3, IGF1, and their combination in predicting NPC metastasis. The combined diagnosis model showed the highest AUC value (0.927), indicating superior predictive performance compared to the individual biomarkers.

ROC curve analysis was performed to evaluate the predictive value of serum VEGFC, VEGFR-3, and IGF1 levels, both individually and in combination, for NPC metastasis. The combined model of the three biomarkers demonstrated a significantly higher AUC (0.927, 95% CI: 0.891–0.963) compared to each marker alone (VEGFC AUC: 0.706; VEGFR-3 AUC: 0.798; IGF1 AUC: 0.695), indicating superior predictive performance (**Table 5**, **Figure 2**).

**Table 5:** ROC curve analysis of serum biomarkers for predicting NPC metastasis.

Biomarker(s)	Optimal Cut-off Value	AUC	95% CI for AUC	Sensitivity	Specificity
VEGFC	>501.5 ng/L	0.706	0.638–0.774	0.731	0.627
VEGFR-3	>1205.1 ng/L	0.798	0.739–0.857	0.782	0.714
IGF1	>82.5 ng/mL	0.695	0.625–0.765	0.667	0.682
Combined (VEGFC+VEGFR-3+IGF1)	–	0.927	0.891–0.963	0.885	0.841

**Figure 3:**
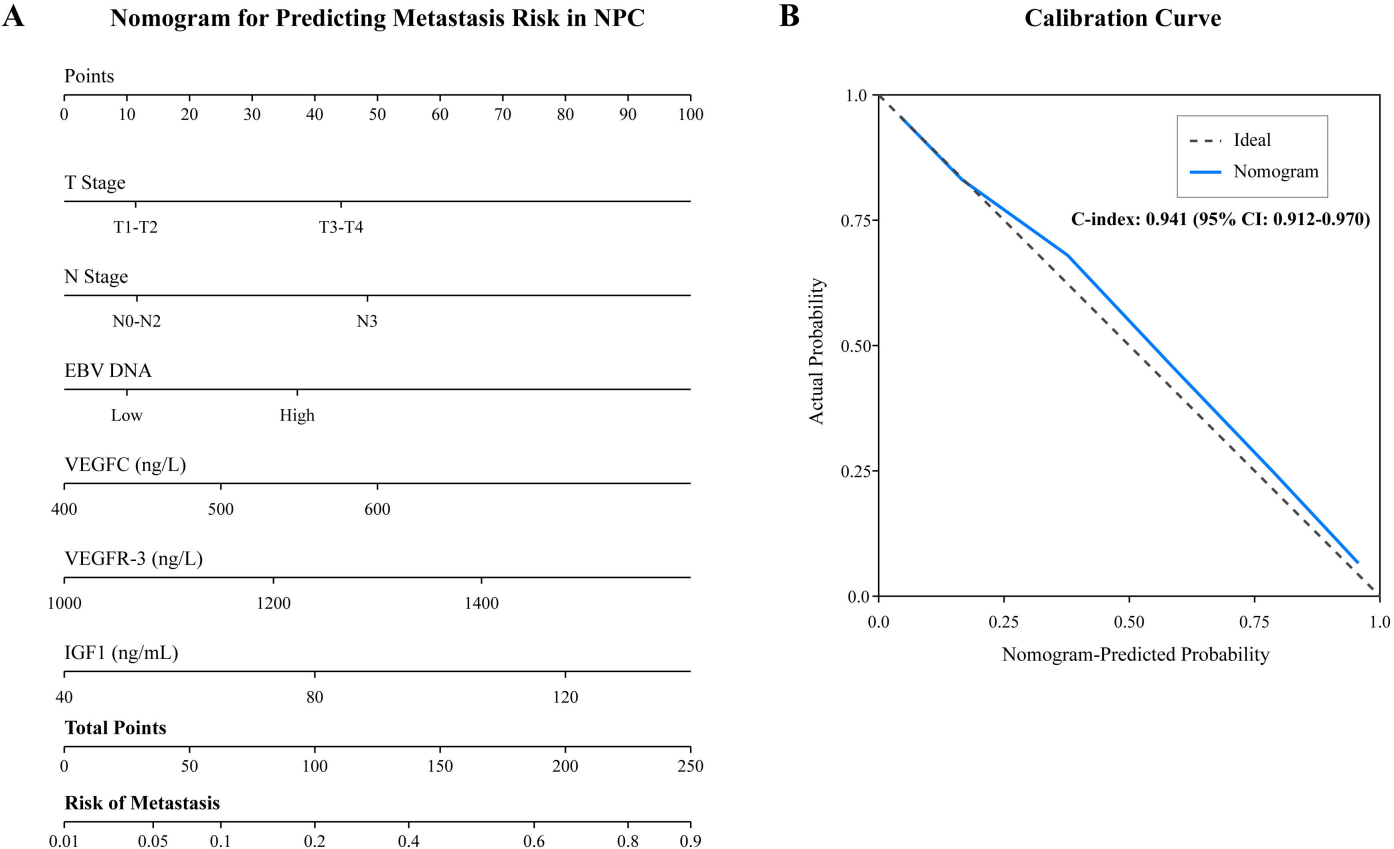
Nomogram for predicting metastasis in NPC patients. **(A)** The nomogram was developed based on six independent risk factors: T stage, N stage, plasma EBV DNA load, and serum levels of VEGFC, VEGFR-3, and IGF1. To use the nomogram, locate the patient’s value for each variable on its axis, draw a vertical line up to the “Points” scale to determine the score for each variable, sum the scores, and locate the total score on the “Total Points” axis. Draw a vertical line down to the “Risk of Metastasis” axis to find the patient’s predicted probability. **(B)** The calibration curve for the nomogram. The x-axis represents the nomogram-predicted probability of metastasis, and the y-axis represents the actual observed metastasis rate. The dashed line represents the ideal prediction, while the solid line represents the performance of the current model.

To provide a clinically applicable tool for individualized risk prediction, a nomogram was constructed incorporating all independent risk factors identified in the multivariate analysis (T stage, N stage, EBV DNA load, VEGFC, VEGFR-3, and IGF1) (**Figure 3**). The nomogram demonstrated excellent discrimination, with a C-index of 0.941 (95% CI: 0.912–0.970). The calibration curve, plotted to assess the agreement between predicted and actual probabilities, showed strong concordance with the ideal 45-degree line, indicating high predictive accuracy (**Figure 3**).

### 3.5 Survival analysis

**Figure 4:**
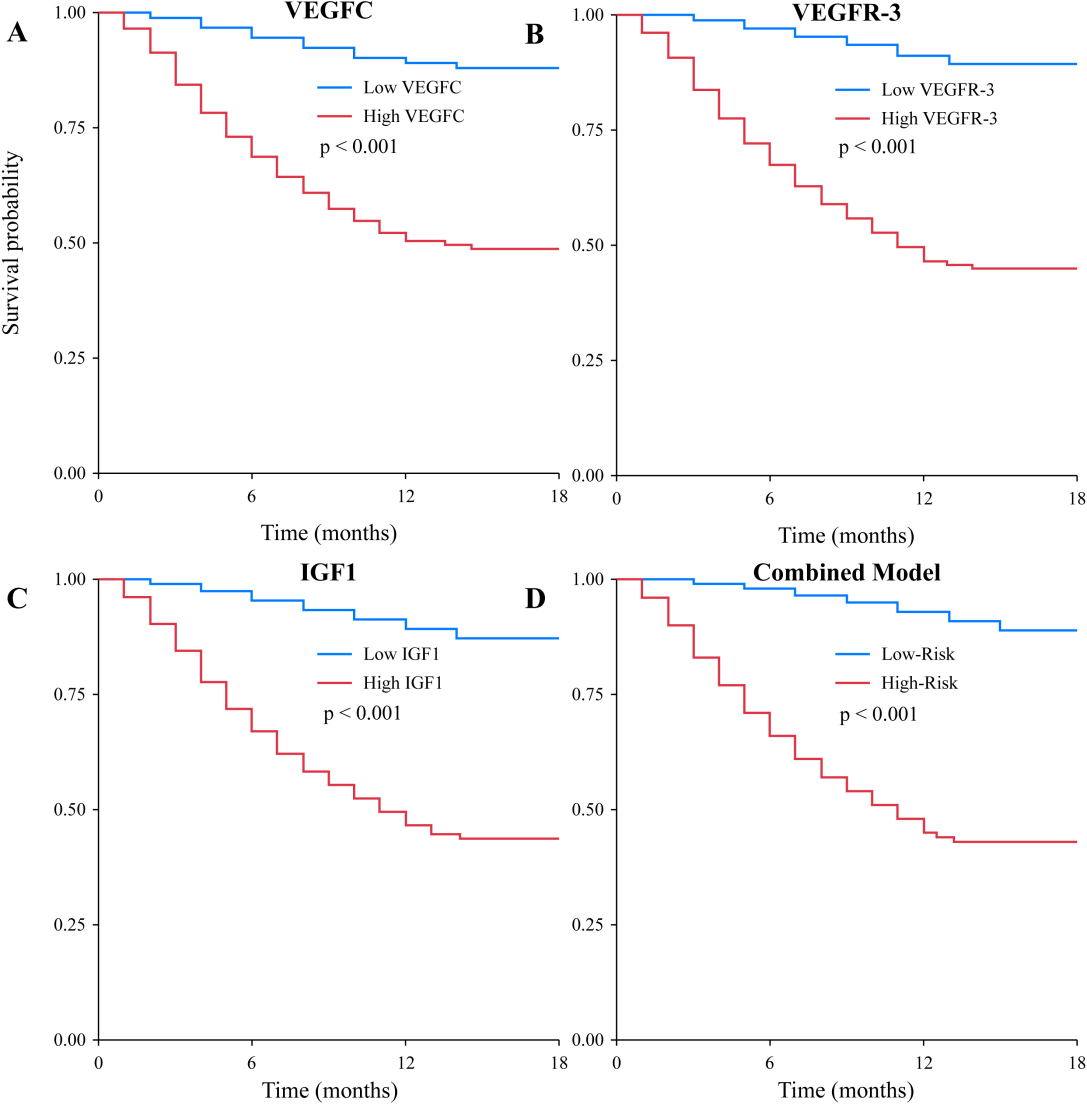
Kaplan-Meier curves for overall survival based on biomarker levels. Survival curves were plotted for patients stratified by **(A)** low vs. high VEGFC levels, **(B)** low vs. high VEGFR-3 levels, **(C)** low vs. high IGF1 levels, and **(D)** low-risk vs. high-risk groups based on the combined three-biomarker model. In all cases, high biomarker levels or a high-risk classification were significantly associated with poorer overall survival (all log-rank P<0.001).

To further investigate the prognostic value of the biomarkers, we performed Kaplan-Meier survival analysis. The results showed that patients with high serum VEGFC levels (>501.5 ng/L) had significantly shorter OS compared to those with low levels (log-rank P<0.001, **Figure 4**). Similarly, high levels of VEGFR-3 (>1205.1 ng/L) and IGF1 (>82.5 ng/mL) were both associated with significantly poorer OS (log-rank P<0.001 for both, **Figures 4B**). The combined biomarker classifier, which integrates all three markers, demonstrated the strongest prognostic stratification. Patients in the high-risk group, as defined by the combined model, exhibited markedly worse OS than those in the low-risk group (log-rank P<0.001, **Figure 4**). Median OS was not reached in the low-risk or low-biomarker groups, while it was significantly shorter in the high-risk and high-biomarker groups (**Table 6**). Furthermore, a subgroup analysis was conducted exclusively on the 78 patients with distant metastasis. Even within this uniformly poor-prognosis cohort, the combined biomarker classifier retained its prognostic power. Metastatic patients classified as having a “low-risk” biomarker profile had a significantly better OS compared to those classified as “high-risk” (median OS: 16.8 months vs. 10.2 months; log-rank P = 0.015).

**Table 6:** Overall survival analysis based on biomarker stratification.

Biomarker Stratification	Group	No. of Patients	No. of Events	Median OS (months)	95% CI for Median OS	Log-rank P-value
VEGFC	Low (≤501.5 ng/L)	183	21	Not Reached	–	<0.001
	High (>501.5 ng/L)	115	68	13.5	11.8–15.2	
VEGFR-3	Low (≤1205.1 ng/L)	169	18	Not Reached	–	<0.001
	High (>1205.1 ng/L)	129	71	12.9	11.0–14.8	
IGF1	Low (≤82.5 ng/mL)	195	25	Not Reached	–	<0.001
	High (>82.5 ng/mL)	103	64	14.1	12.2–16.0	
Combined Biomarker Model	Low-Risk	198	22	Not Reached	–	<0.001
	High-Risk	100	67	12.4	10.7–14.1	

## 4 Discussion

Nasopharyngeal carcinoma, an Epstein-Barr virus-associated malignancy with a high prevalence in specific geographic regions, often presents at an advanced stage, leading to poor prognosis (1, 16). Despite advances in treatment modalities, distant metastasis remains a major challenge. This study investigated the clinical significance of serum VEGFC, VEGFR-3, and IGF1 levels as biomarkers for metastasis and prognosis in NPC patients.

Our findings revealed that elevated serum levels of VEGFC, VEGFR-3, and IGF1 were significantly associated with both the presence of metastasis and poor prognosis in NPC patients. This is consistent with the known biological roles of these molecules. VEGFC, by binding to VEGFR-3, is a potent inducer of lymphangiogenesis, creating pathways for tumor cell dissemination (17, 18). Increased VEGFC/VEGFR-3 signaling has been linked to lymphatic metastasis in various cancers, including recent reports in pancreatic (19) and breast cancer (20, 21). The higher levels of these markers in metastatic NPC patients observed in our study suggest an active lymphangiogenic process contributing to tumor spread.

IGF1 is a crucial component of the IGF signaling system, which plays a pivotal role in cell growth, survival, and metabolism (22). Dysregulation of the IGF1 pathway has been implicated in tumorigenesis and progression across multiple cancer types by promoting cell proliferation and inhibiting apoptosis (23, 24). Our data, showing elevated IGF1 in NPC patients with metastasis and poor prognosis, align with studies suggesting that IGF1 can enhance tumor invasiveness. This suggests IGF1 may create a favorable microenvironment for tumor development by inhibiting apoptosis and promoting growth, a mechanism also observed in other malignancies (25). While previous research has focused on IGF1 in cancers like gastric and ovarian cancer (23, 26), its role in NPC is becoming increasingly recognized (27).

The study also identified advanced T stage, N stage, high EBV DNA load, and elevated serum VEGFC, VEGFR-3, and IGF1 as independent risk factors for NPC metastasis. Clinical stage, particularly T and N classifications, inherently reflects tumor burden and extent of spread (28). High plasma EBV DNA load is a well-established adverse prognostic factor in NPC, reflecting tumor burden and activity (29). Our finding that high EBV DNA is an independent predictor of metastasis is consistent with contemporary literature (30). The independent predictive value of VEGFC, VEGFR-3, and IGF1, even after adjusting for these strong clinical predictors, underscores their potential as valuable molecular biomarkers. High VEGFC levels may reflect an increased capacity for distant metastasis, as suggested by studies in other cancers, which have shown that VEGFC and VEGFR-3 expression correlate with lymph node metastasis and poor outcomes (31). Similarly, a dysregulated IGF1 axis has been shown to predict unfavorable survival in NPC patients (32).

Furthermore, our ROC curve analysis and the newly developed nomogram highlight the power of a multi-marker approach. The combined biomarker panel had superior predictive accuracy for metastasis compared to individual markers. The nomogram, which integrates these biomarkers with clinical factors and EBV DNA, provides a quantitative and individualized risk assessment tool. Such models are increasingly recognized for their ability to translate complex statistical data into clinically actionable information, helping to identify high-risk patients who might benefit from more aggressive treatment or intensive surveillance (33, 34). In addition to predicting metastasis, our study demonstrates the robust prognostic value of these biomarkers for overall survival. The Kaplan-Meier analysis confirmed that high levels of each biomarker, and particularly a high-risk score from the combined classifier, were strongly associated with decreased survival time. This finding aligns with a recent meta-analysis which suggested that a multi-marker panel, incorporating indicators of angiogenesis, cell proliferation, and viral load, offered superior prognostic accuracy for survival in NPC compared to single markers (35, 36). Crucially, our subgroup analysis of metastatic patients revealed that the combined biomarker score could further stratify survival even within this advanced-stage group. This suggests that the biological profile reflected by these serum markers may indicate a more aggressive tumor phenotype, independent of the mere presence of metastases, which could have implications for selecting patients for more aggressive systemic therapies or novel targeted agents. This integrated approach aligns with the principles of precision oncology, aiming to tailor patient management based on a comprehensive risk profile.

This study has several limitations. Firstly, its retrospective design may be subject to selection bias and unmeasured confounding variables. Secondly, it was conducted at a single institution, which might limit the generalizability of the findings to other populations. Thirdly, while we identified associations, the precise molecular mechanisms linking these serum markers to NPC progression require further elucidation through functional studies. Fourthly, the study lacked a non-cancer or benign disease control group, which limits our ability to assess the specificity of these biomarkers for NPC against other conditions. Future studies should include control groups to establish more robust diagnostic and prognostic specificity. Future prospective, multicenter studies with larger sample sizes are warranted to validate these findings, including the clinical utility of the nomogram, and to explore the dynamic changes in these biomarkers during treatment and their correlation with treatment response.

## 5 Conclusion

In conclusion, this study demonstrates that elevated serum levels of VEGFC, VEGFR-3, and IGF1 are significantly associated with metastasis and poor prognosis in patients with nasopharyngeal carcinoma. Advanced T stage, N stage, high EBV DNA load, and high levels of these three biomarkers were identified as independent risk factors for NPC metastasis. The combined assessment of these biomarkers offers enhanced predictive value for both metastatic risk and overall survival, and the developed nomogram provides a practical tool for individualized risk stratification. These findings suggest that VEGFC, VEGFR-3, and IGF1, integrated with clinical factors and EBV status, hold promise as non-invasive tools for risk assessment in NPC, potentially guiding personalized treatment approaches.

## Data Availability

The original contributions presented in the study are included in the article/**Supplementary Material**. Further inquiries can be directed to the corresponding author.
