# Supplementary material for: Association of serum vascular endothelial growth factor-C, vascular endothelial growth factor receptor-3, and insulin-like growth factor 1 levels with metastasis and prognosis in patients with nasopharyngeal carcinoma
Source: Front Oncol. 2025 Nov 17;15:1655015. doi: 10.3389/fonc.2025.1655015 (PMC12665541; doi:10.3389/fonc.2025.1655015)
Supplement: Supplementary file 1 [file Table1.docx]

**Supplementary Table S1. Correlation between Serum Biomarkers and Plasma EBV DNA Load**

|  | **VEGFC** | | **VEGFR-3** | | **IGF1** | |
| --- | --- | --- | --- | --- | --- | --- |
| **Variable** | **Spearman's ρ** | **P-value** | **Spearman's ρ** | **P-value** | **Spearman's ρ** | **P-value** |
| EBV DNA Load (log-transformed) | 0.485 | <0.001 | 0.512 | <0.001 | 0.466 | <0.001 |
